# Pre-hospital Emergency Services Survey in traffic accidents in Isfahan Province 

**Published:** 2019-08

**Authors:** Reihaneh Rastegari, Leyla Alsafi, Mohsen Eftekhari

**Affiliations:** ^*a*^Pre-hospital Emergency Department, Isfahan University of Medical Sciences, Isfahan, Iran.

## Abstract

**Background:**

Considering the importance of the role and function of pre-hospital emergency and community health and the need for continuous monitoring of traffic accidents and the process of service to patients, the present study was conducted to investigate the pre-hospital emergency services of the cities of Isfahan during one year in traffic accidents

**Methods:**

This study was a descriptive-cross-sectional study. The statistical population consisted of all traumatic victims injured in the emergency operation area of 115 provinces of Isfahan from April 1, 1997 to the end of February. The files were 61994 cases. Data were recorded by the Esfahan Emergency Department s Mission Information Registration Form E-patients were extracted and analyzed using SPSS18 software.

**Results:**

Of the total number of missions in 22 provinces of Isfahan province, 44.2% belonged to Isfahan and then Najafabad city was 4.8%, and the cities of Khomeini were 7.7% and Lenjan, 7% of the missions. 78% of the missions resulted in admission, 11% outpatient, 10% abandonment of the mission and 1% infidelity, which was examined separately and comparatively.

**Conclusions:**

Improving the training and skills of pre-hospital emergency staff and culture-building, and changing the society s attitude toward receiving its services is the key to improving the status quo.

**Keywords:**

Pre-hospital Emergency, Traffic Accidents, Isfahan Province

